# Supplementary material for: COZOID: contact zone identifier for visual analysis of protein-protein interactions
Source: BMC Bioinformatics. 2018 Apr 6;19:125. doi: 10.1186/s12859-018-2113-6 (PMC5889581; doi:10.1186/s12859-018-2113-6)
Supplement: Supplementary file 2 — Software build. Executable binary file of the software tool. (ZIP 118,784 kb) [file 12859_2018_2113_MOESM2_ESM.zip › cozoid/java/docs/beansbinding-1.2.1-doc.zip!/org/jdesktop/beansbinding/class-use/BindingListener.html]

Uses of Interface org.jdesktop.beansbinding.BindingListener (Beans Binding)


---


|  |  |  |
| --- | --- | --- |
| **Overview** \| **Package** \| **Class** \| **Use** \| **Tree** \| **Deprecated** \| **Index** \| **Help** | |  |
| PREV   NEXT | **FRAMES**    **NO FRAMES**     **All Classes** |  |


---


## **Uses of Interface org.jdesktop.beansbinding.BindingListener**

| Packages that use BindingListener | |
| --- | --- |
| **org.jdesktop.beansbinding** | Provides support for defining properties and creating bindings between sets of two properties. |

Uses of BindingListener in org.jdesktop.beansbinding

| Classes in org.jdesktop.beansbinding that implement BindingListener | |
| --- | --- |
| `class` | `AbstractBindingListener`             An abstract subclass of `BindingListener` that simplifies writing `BindingListeners` by allowing you to extend this class and re-implement only the methods you care about. |

| Methods in org.jdesktop.beansbinding that return BindingListener | |
| --- | --- |
| `BindingListener[]` | `BindingGroup.getBindingListeners()`             Returns the list of `BindingListeners` registered on this group. |
| `BindingListener[]` | `Binding.getBindingListeners()`             Returns the list of `BindingListeners` registered on this `Binding`. |

| Methods in org.jdesktop.beansbinding with parameters of type BindingListener | |
| --- | --- |
| `void` | `BindingGroup.addBindingListener(BindingListener listener)`             Adds a `BindingListener` to be notified of all `BindingListener` notifications fired by any `Binding` in the group. |
| `void` | `Binding.addBindingListener(BindingListener listener)`             Adds a `BindingListener` to be notified of changes to this `Binding`. |
| `void` | `BindingGroup.removeBindingListener(BindingListener listener)`             Removes a `BindingListener` from the group. |
| `void` | `Binding.removeBindingListener(BindingListener listener)`             Removes a `BindingListener` from the `Binding`. |

---


|  |  |  |
| --- | --- | --- |
| **Overview** \| **Package** \| **Class** \| **Use** \| **Tree** \| **Deprecated** \| **Index** \| **Help** | |  |
| PREV   NEXT | **FRAMES**    **NO FRAMES**     **All Classes** |  |


---
